# Agro-morphological, biochemical, and molecular markers of barley genotypes grown under salinity stress conditions

**DOI:** 10.1186/s12870-023-04550-y

**Published:** 2023-10-30

**Authors:** Marwa M. Ghonaim, A. M. Attya, Heba G. Aly, Heba I. Mohamed, Ahmed A. A. Omran

**Affiliations:** 1 Cell Study Research Department, Field Crops Research Institute, Agriculture Research Center, Giza, Egypt; 2 Barley Research Department, Field Crops Research Institute, Agriculture Research Center, Giza, Egypt; 3 Faculty of Education, Biological and Geological Sciences Department, Ain Shams University, El Makres St. Roxy, Cairo, 11341 Egypt

**Keywords:** Barley, Salinity stress, Flavonoid, SDS-PAGE, SCoT, ISSR, UPGMA

## Abstract

The aim of this study was to evaluate the impact of salt stress on morphological, yield, biochemical, and molecular attributes of different barley genotypes. Ten genotypes were cultivated at Fayoum Research Station, El-Fayoum Governorate, Egypt, during two seasons (2020–2021 and 2021–2022), and they were exposed to two different salt concentrations (tap water as a control and 8000 ppm). The results showed that genotypes and salt stress had a significant impact on all morphological and physiological parameters. The morphological parameters (plant height) and yield attributes (spike length, number of grains per spike, and grain yield per plant) of all barley genotypes were significantly decreased under salt stress as compared to control plants. Under salt stress, the total soluble sugars, proline, total phenol, total flavonoid, ascorbic acid, malondialdehyde, hydrogen peroxide, and sodium contents of the shoots of all barley genotypes significantly increased while the potassium content decreased. L1, which is considered a sensitive genotype was more affected by salinity stress than the tolerance genotypes L4, L6, L9, and Giza 138. SDS-PAGE of seed proteins demonstrated high levels of genetic variety with a polymorphism rate of 42.11%. All genotypes evaluated revealed significant variations in the seed protein biochemical markers, with new protein bands appearing and other protein bands disappearing in the protein patterns of genotypes cultivated under various conditions. Two molecular marker techniques (SCoT and ISSR primers) were used in this study. Ten Start Codon Targeted (SCoT) primers exhibited a total of 94 fragments with sizes ranging from 1800 base pairs to 100 base pairs; 29 of them were monomorphic, and 65 bands, with a polymorphism of 62.18%, were polymorphic. These bands contained 21 unique bands (9 positive specific markers and 12 negative specific markers). A total of 54 amplified bands with molecular sizes ranging from 2200 to 200 bp were produced using seven Inter Simple Sequence Repeat (ISSR) primers; 31 of them were monomorphic bands and 23 polymorphic bands had a 40.9% polymorphism. The techniques identified molecular genetic markers associated with salt tolerance in barley crop and successfully marked each genotype with distinct bands. The ten genotypes were sorted into two main groups by the unweighted pair group method of arithmetic averages (UPGMA) cluster analysis based on molecular markers and data at a genetic similarity coefficient level of 0.71.

**Supplementary Information:**

The online version contains supplementary material available at 10.1186/s12870-023-04550-y.

## Introduction

The salinity of the soil and water has recently become a serious global issue, limiting the growth, production, and overall quality of most crops [1]. Salinity stress is one of the major abiotic stressors that reduces plant growth and causes yield losses [2]. Because of global climate change and a decrease in the quantity and quality of irrigation water, more land will be affected during the next few decades [3]. By 2050, it's expected that salinity will have a negative impact on more than 50% of agricultural land [3]. Around 20% of all cultivated land and 33% of all irrigated agricultural regions suffer from high salinity globally [4]. Agronomic practices, soil composition, and other environmental factors have an important effect on the technological quality criteria for barley. Saline water is used in agriculture, which harms the soil and has a negative impact on crop quality and productivity. Due to this problem, it is essential to find and create resilient crops that can cope with salinity stress [5]. The environment, which is characterised by an increasing water deficit and temperature stress during grain filling, may cause major changes in grain production as well as grain quality features, particularly protein content [6]. This effect is most pronounced in Mediterranean climates [6].

There are two hypotheses that explain how salt inhibits plant growth: either by ion toxicity or by disrupting osmotic processes [7]. Salinity inhibited a number of physiological processes in plants, including their ability to absorb water [8], reduce nutrient absorption [9], decrease photosynthetic rate [10], and lower yield productivity [11]. Due to the accumulation of sodium ions, which promote the generation of reactive oxygen species (ROS) like the hydroxyl radical, singlet oxygen, hydrogen peroxide, and superoxide, salinity causes osmotic stress and ionic toxicity [12]. This causes oxidative stress, which damages mitochondria, chloroplasts, the functional structure of cells, biomolecules including lipids, proteins, and nucleic acids, etc., leading to lipid peroxidation, protein denaturation, and DNA mutation in plant cells [13, 14]. Therefore, plants can produce antioxidants to scavenge ROS [15]. In addition, soluble sugar, proline, and phenolic substances are thought to be crucial indicators of a plant's capacity to tolerate salt stress and they also accumulate in response to abiotic stress [16, 17]. Osmolytes act as antioxidants, buffer the cellular redox potential, stabilize membranes and macromolecules, and serve as sources of energy during stress recovery, maintaining the functional equilibrium of the cell and protecting plant cells [17]. Moreover, protein accumulates in plants exposed to salt stress and may serve as energy storage or regulate the osmotic potential [11].

Evaluation of genetic diversity within a gene pool improves genotype selection, stimulates the best genetic advancement, and reduces breeding time [18]. Genetic markers have become the approved and widely used method for documenting rare plants [19]. Plant genetic diversity is effectively assessed using inter-simple sequence repeats (ISSR) and start codon targeted (SCoT) markers, which may also serve as markers for different characteristics including salinity tolerance [20]. DNA fingerprinting, QTL mapping, cultivar identification, and other genetic techniques all use SCoT markers [21]. The short conserved area bordering the ATG start codon in plant genes serves as the foundation for the SCoT marker, which is a dominant and repeatable marker [20]. This kind of marker may be crucial for genotyping wheat and finding polymorphisms [22]. It also has a number of benefits over RAPD, ISSR, and AFLP, including being more stable, providing bands that are reproducible and reliable, and being suitable for population research; genetic mapping in a variety of plants, and marker-assisted selection programs [11]. Furthermore, ISSR markers are among the most potent marker systems now in use, creating a variety of informational bands [23]. ISSR markers are frequently used since they are thought to be able to amplify DNA areas between two microsatellites [24].

Because they employ random markers and may be produced without precise sequence information, ISSRs provide a good example of the selectivity of microsatellite markers [25]. ISSR primers are potent molecular markers that can distinguish between genotypes due to their variation in polymorphism, resolving power (Rp), and the informativeness of the bands (Ib) [26]. Any plant species can be employed if the genome has enough ISSR motifs [27]. The ISSR marker can also be used in barley genotypes to accurately quantify genetic variation and population structure [28, 29].

*Hordeum vulgare* L., (barley) is a crop with a wide range of adaptability and a brief growing season. It belongs to the grass family and has 14 chromosomes. After wheat, rice, and corn, barley is the fourth most significant cereal in terms of global agricultural production [30]. It is considered the fourth-ranked cereal crop and one of the most significant essential grains grown in many developing countries is barley [31]. Although barley has a modest tolerance for salinity, its morpho-physiological characteristics and yield characteristics have received the least attention on degraded marginal soils. It is a fantastic model crop for research into the genetics and mechanisms behind salt tolerance, which may be used to create methods to increase salt tolerance in cereals [32].

In order to maintain yield in extremely dry and salty areas, it has therefore been suggested to cultivate salt-tolerant barley genotypes. Breeding is crucial for creating toolkits of salt-tolerant crop genotypes and for implementing growth, physiological, and yield features in the field [33]. According to El-Khalifa et al. [34], barley is an essential crop in Egypt and takes up 76.9% of freshly reclaimed land. Also, Mohamed et al. [14] reported that, barley productivity in Egypt reached 108,000 tons in 2019 and is predicted to continue rising. Barley can be used as animal feed, to make bread, and to make a variety of healthful foods [35].

The objective of this study is to identify the most salt-tolerant barley genotypes and enhance breeding programs aimed at boosting production by examining the variations of the ten different barley genotypes using biochemical and molecular markers (SCoT and ISSR), as well as screening and analyzing the degree of variance in yield traits among different barley genotypes.

## Materials and methods

### Plant materials

Ten genotypes of barley served as the study's experimental materials. These genotypes consist of the check variety Giza 138 and nine lines (L1, L2, L3, L4, L5, L6, L7, L8, and L9). Table 1 provides the name, pedigree, and origin of the genotypes under study. Healthy grains of the ten barley genotypes were obtained fromthe barley Research Department, Field Crop Research Institute, Agricultural Research Centre, Giza, Egypt. Two field experiments were carried out at Fayoum Research Station, El-Fayoum Governorate, Egypt, during the successive seasons (2020–2021 and 2021–2022). Each experiment consisted of three replicates of the Randomized Complete Block Design (RCBD), with one genotype planted on each experiment plot in four rows that were each 3 m long and spaced 20 cm apart (the plot area was 1.6 m_2_). Both experiments were in the isometer; the first experiment was irrigated by tap water and the other received 8000 ppm salt water. The mechanical and chemical analysis of the experimental soil is presented in Table 2 provides a description of the experimental site along with a soil analysis.

**Table 1:** Pedigree, name and seed origin of barley genotypes

Name	Pedigree	Origin
L1	Giza 138/Lignee527/NK1272//JLB70-063/3/Rhn-03/4/ICB_116132	**ICARDA/ Egypt**
L2	Giza 138 /Carbo/Hamra/4/Rhn-08/3/DeirAlla106//DL71/ Strain205/5/Aths/Lignee686/4/Avt/Attiki//Aths/3/Giza121/Pue	**ICARDA/ Egypt**
L3	Giza 138/ Rihane-03/3/As46/Aths*2//Aths/Lignee686/6/Rhn-03/Eldorado/5/Rhn-03//Lignee527/NK1272/4/Lignee527/Chn-01/3/Alanda	**ICARDA/ Egypt**
L4	Giza 138/ Melusine/Aleli/3/Matico/Jet//Shyri/4/Canela/5/ Lignee527/NK1272//JLB70-063/3/Rhn-03	**ICARDA/ Egypt**
L5	Giza 138/ Rhn-03/Eldorado/5/Rhn-03// Lignee527/ NK1272/4/ Lignee527/Chn-01/3/Alanda/ 6/Aths/Lignee686/ 4/Avt/Attiki// Aths/3/Giza121/Pue	**ICARDA/ Egypt**
L6	Giza 138/ Mtn-01/Bda	**ICARDA/ Egypt**
L7	Giza 138/ ICNB93-369/3/Moroc9-75//WI2291/WI2269	**ICARDA/ Egypt**
L8	Giza 138/ Rhn-03/Eldorado/5/Rhn-03//Lignee527/NK1272/4/Lignee527/Chn-01/3/Alanda/6/Aths/Lignee686/4/Avt/Attiki//Aths/3/Giza121/Pue	**ICARDA/ Egypt**
L9	Giza 138/ Rhn//Bc/Coho/3/DeirAlla106//Api/EB89-8–2-15–4/5/CM67/3/Apro//Sv02109/Mari/4/Carbo/6/Lignee527/NK1272//JLB70-063/3/Rhn-03	**ICARDA/ Egypt**
Giza 138	Acsad1164/3/Mari/Aths*2//M-Att-73–337-1/5/Aths/lignee686/3/Deir Alla106//Sv.Asa/Attiki/4/Cen/Bglo.”S”	**CHECK**

**Table 2:** Mechanical and chemical analysis of location’s soil

Location	Available (ppm)			pH	EC dS/m	CaCO_3_%	Clay %	Silt %	Sand %	Soil texture
	**N**	**P**	**K**							
El-Fayoum	95	7.8	0.75	8.3	11.6	0.73	50.8	39.2	10	Clay Loam

These analyses were done by Soil and Water Research Institute, ARC, Egypt

### Irrigation water and salinity levels

The anions and cations content in tap and saline water was illustrated in Table 3.

**Table 3:** Content of anions and cations in tap and saline water

Treatments	Cations (mg/l)			pH	Salt concentration ppm	K + ppm	Cl- ppm	SO_4_^2^- ppm
	**Ca**^**++**^	**Mg**^**++**^	**Na + **					
**Tap water**	40	75	33	7.6	1000	0.28	3.55	6.91
**Saline water**	37	75	38	8.2	8000	0.65	6.65	25.06

These analyses were done by Soil and Water Research Institute, ARC, Egypt

### Studied characters

To differentiate between the studied genotypes based on morphological characteristics, the following parameters were recorded: plant height (cm), spike length (cm), number of grains per spike and grain yield per plant (g).

### Salinity tolerance indices

The following equations were used to determine the salinity tolerance indices:

Stress susceptibility index (SSI) $=\frac{1-(\frac{\mathrm{Ys}}{\mathrm{Y}})}{1-({\overset{-}{\text{Y}}}\mathrm{s }/{\overset{-}{\text{Y}}}\mathrm{p})}$ [36].

Stress tolerance index (STI) $=\frac{(\mathrm{Yp})(\mathrm{Ys})}{({\overset{-}{\text{Y}}}\mathrm{p})2}$ [37].

Mean productivity (MP) $=\frac{\mathrm{YP }+\mathrm{YS}}{2}$ [38].

Tolerance TOL = YP − YS [38].

Yield index (YI) $=\frac{\mathrm{Ys}}{{\overset{-}{\text{Y}}}\mathrm{s}}$ [39].

Yield stability index (YSI) $=\frac{\mathrm{Ys}}{\mathrm{YP}}$ [40].

YS and YP are the mean yields of each genotype under stress and non-stress conditions, respectively.

Ȳs and Ȳp are the mean yields of all genotypes under stress and non-stress conditions, respectively.

### Determination of osmolytes contents

Soluble sugars were measured in the shoots of barley genotypes using the phenol–sulphuric method according to Dubois et al. [41] method. In test tubes, 1 mL of the supernatant was combined with 5 mL of concentrated sulfuric acid and 1 mL of a 5% phenol solution. After vortexing, the tubes were allowed to cool for five minutes before the optical density at 485 nm was determined. The soluble sugar levels were compared with a standard curve of glucose. According to Bates [42] methodology, a known weight of dried shoots of barley genotypes was combined with 3% aqueous sulfosalicylic acid (w/v), and the combination was then filtered through filter paper No. 1,and then ninhydrin reagent and acetic acid were added. The absorbance of the mixture was measured at 520 nm using a spectrophotometer.

### Determination of secondary metabolites content

The total phenolic content of dried shoots of barley genotypes was extracted in 80% cold methanol (v/v) for three times at 90ºC. After filtration, the absorbance of the filtrate was recorded at 760 nm by using the Folin-Ciocalteu reagent according to the Dihazi et al. [43] method. Total phenolic content was expressed as µg gallic acid/g DW by using the standard curve of gallic acid. Also, for the determination of flavonoid content, the filtrate was mixed with NaNO_2_ and AlCl_3_ (10% w/v), and the absorbance of the mixture was measured at 510 nm according to the protocol of Bushra et al. [44] using a spectrophotometer. The total flavonoid content was expressed as µg querectin/g DW using the standard curve of querectin.

### Determination of ascorbic acid content

To measure the ascorbic acid content, fresh shoots of different barley genotypes weighing a specified amount (0.5 g) were crushed in 5 ml of 6% trichloroacetic acid (TCA) and centrifuged at 10,000 × g for 20 min. The absorbance of the filtrate was measured at 525 nm using a spectrophotometer after the addition of 2% dinitrophenyl-hydrazine, thiourea, and H_2_SO_4_ as described by Mukherjee and Choudhuri [45] protocol.

### Determination of oxidative stress content

Malondialdehyde content was determined as described by Heath and Packer [46] method to measure the amount of lipid peroxidation. Fresh shoots of barley genotypes were ground in 0.1% trichloroacetic acid and centrifuged at 10,000 × g for 15 min. The supernatant and thiobarbituric acid were mixed together, and the mixture was heated at 95 °C for about 30 min. The non-specific absorbance at 600 nm was removed from the absorption at 532 nm. Using an extinction coefficient of 155 mM^−1^cm^−1^, the malondialdehyde absorbance coefficient was calculated.

A known weight (0.5 g) of fresh barley shoots was crushed in phosphate buffer (50 mM, pH 6.5). The homogenized material was centrifuged at 8000 × g for 20 min. The supernatant was combined with 0.1% titanium sulphate in 20% H_2_SO_4_ and the optical density of the supernatant was measured at 410 nm using a spectrophotometer according to Velikova et al. [47] method to determine H_2_O_2_.

### Determination of minerals

Shoots of barley genotypes were dried at 70°C for 24 h. The dry samples were crushed into an extremely fine powder using a crusher and pestle. Samples (1 g) were dried in crucibles, and placed in an electric oven set to 600 °C and 5 ml of 2N HCl was added to the ash after cooling for 4 h. This solution was then dissolved using boiling deionized water and completed to a volume of 50 ml. The method of Goudarzi and Pakniyat [48] was used to measure K^+^ and Na^+^ using a flame photometer, and the K^+^/Na^+^ ratio was calculated.

### SDS-PAGE analysis

The method described by Laemmli [49] and modified by Studier [50] was used to determine the protein electrophoresis pattern of harvested seeds on 10% polyacrylamide gels. Gel was fixed and stained with 0.25% (w/v) Coomassie Brilliant Blue R-250 following electrophoresis. Using the Gel Doc 2000 Bio-Rad system, the gel was captured on camera, scanned, and examined.

### Genomic DNA extraction

According to https://primerdigital.com/dna.html, genomic DNA was isolated from young barley control leaves. 2% CTAB, 1.5 M NaCl, 10 mM Na_3_EDTA, 0.1 M HEPES-acid, Chloroform-isoamyl alcohol mix (24:1), 100% isopropanol (isopropyl alcohol, 2-propanol), 70% ethanol, and 1xTE (10 mM of Tris-HC1, pH 8.0; 1 mM of EDTA) were used to make the CTAB solution.

### PCR Amplification of ISSR and SCoT Markers

In this investigation, 17 primers (7 ISSR and 10 SCOT) were employed (Table 4). PCR was carried out in 20 μL for both markers containing 10X PCR buffer, 25 mM MgCl_2_, 10 mM dNTPs, 2 μM primer, 5 U Taq DNA polymerase and 100 ng template DNA. All PCR reactions were carried out in an Eppendorf and Perkin Elmer Thermal Cycler. PCR programmed: 95 ºC for 5 min; 35 cycles (95ºC for 30 s, Tm ºC for 45 s, 72ºC for 1:30 min) and 72ºC for 5 min.

**Table 4:** List of ISSR and SCOT primers and their sequence and melting temperature (Tm)

Primer cod	Sequence	Tm
SCOT 21	ACGACATGGCGACCCACA	61
SCOT 22	AACCATGGCTACCACCAC	65
SCOT 23	CACCATGGCTACCACCAG	61
SCOT 24	CACCATGGCTACCACCAT	65
SCOT 25	ACCATGGCTACCACCGGG	67
SCOT 26	ACCATGGCTACCACCGTC	61
SCOT 27	ACCATGGCTACCACCGTG	61
SCOT 28	CCATGGCTACCACCGCCA	67
SCOT 29	CCATGGCTACCACCGGCC	72
SCOT 31	CCATGGCTACCACCGCCT	67
ISSR-807	AGAGAGAGAGAGAGAGT	50
ISSR-810	GAGAGAGAGAGAGAGAT	50
ISSR-835	AGAGAGAGAGAGAGAGYC	55
ISSR-841	GAGAGAGAGAGAGAGAYC	55
UBC826	ACACACACACACACACC	52
UBC827	ACACACACACACACACG	52
UBC835	AGAGAGAGAGAGAGAGYC	55

Electrophoresis was used to separate the amplification products in a 1.2% agarose gel containing ethidium bromide (0.5 µg/mL) in 1X THE buffer at 80 V.PCR products were photographed and observed under UV light using a Gel Documentation System (BIO-RAD 2000).

### Data analysis

For each ISSR and SCoT marker study, the band profiles were only rated as present (1) or absent (0) for distinct, reproducible bands. Ten SCoT and seven ISSR primers' banding patterns were evaluated in order to determine the degree of genetic relatedness between the samples under investigation. The information obtained from ISSR and SCoT was analyzed using binary values (1, 0), and the results were used to generate a phenogram that deals with the genetic links among the genotypes investigated. The similarity coefficients were computed using Jaccard's coefficient and the Unweighted Pair Group Method with Arithmetic Averages (UPGMA) and SAHN (Sequential, Agglomerative, Hierarchical, and Nested Clustering) algorithms from the NTSYS-PC (Numerical Taxonomy and Multivariate Analysis System), version 2.1 (Applied Biostatistics) program [51]. In addition, using PAST software 4.02 (https://www.nhm.uio.no/english/research/infrastructure/past/), a principal component analysis (PCA) based on the ISSR and SCoT data matrix was created. ClustVis, a web tool for visualizing clustering of multivariate data, was used to construct heat maps (https://biit.cs.ut.ee/clustvis/) [52].

### Statistical analysis

For agro-morphological, and biochemical data, the analysis of variance (ANOVA) was conducted independently for each treatment and then a homogeneity test of errors was checked before conducting a seasonal combined analysis. According to the method described by Gomez and Gomez [53], the significance of mean performances was assessed for all measured agro-morphological and physiological variables using Duncan's multiple range test [54].

## Results

### Effect of salinity stress on agro-morphological traits

The results of the ten barley genotypes tested under salinity stress (8000 ppm) during the two successive seasons are shown in Table 5. All barley genotypes showed a substantial drop in morphological parameters (plant height) and yield attributes (spike length, number of grains per spike, and grain weight per plant) as compared to their unstressed plants.

**Table 5:** Effect of salt stress on morphological parameters and yield attributes of barley genotypes during two successive seasons (2020–2021 and 2021–2022)

Genotypes	Plant height (cm)		Spike length (cm)		Number of grain per spikes		Grain yield per plant (g)	
	**Control**	**Salt stress**	**Control**	**Salt stress**	**Control**	**Salt stress**	**Control**	**Salt stress**
**L1**	74.0 ± 1.5^ef^	65.0 ± 1.5^h^	9.7 ± 0.4^bc^	6.7 ± 0.2^h^	46.0 ± 0.8^d^	33.0 ± 0.5^h^	5.6 ± 0.2^c^	2.5 ± 0.05^f^
**L2**	82.0 ± 2.0^cd^	69.0 ± 1.2^g^	9.5 ± 0.4^c^	7.5 ± 0.2^f^	45.5 ± 0.9^d^	36.5 ± 0.5^g^	7.0 ± 0.2^a^	4.0 ± 0.1^e^
**L3**	88.0 ± 2.2^b^	75.0 ± 1.5^e^	10.0 ± 0.5^b^	7.5 ± 0.2^f^	49.0 ± 0.8^b^	43.0 ± 0.6^e^	6.6 ± 0.2^ab^	3.9 ± 0.05^e^
**L4**	77.5 ± 1.7^e^	66.0 ± 1.0^h^	10.5 ± 0.5^a^	8.7 ± 0.4^d^	52.5 ± 1.1^a^	40.0 ± 0.5^f^	5.7 ± 0.1^c^	4.9 ± 0.2^d^
**L5**	72.5 ± 1.5^f^	64.5 ± 1.1^h^	8.7 ± 0.3^d^	7.5 ± 0.3^f^	47.0 ± 1.0^cd^	39.5 ± 0.4^f^	6.7 ± 0.3^a^	3.7 ± 0.1^e^
**L6**	91.0 ± 2.5^a^	73.0 ± 1.6^f^	10.2 ± 0.6^a^	8.5 ± 0.4^d^	52.5 ± 1.3^a^	40.0 ± 0.7^f^	5.3 ± 0.1^cd^	4.7 ± 0.2^d^
**L7**	83.0 ± 1.9^c^	71.5 ± 1.7^fg^	9.5 ± 0.4^c^	7.2 ± 0.3^g^	47.0 ± 0.9^c^	37.5 ± 0.^6f^	6.3 ± 0.2^b^	3.7 ± 0.1^e^
**L8**	88.5 ± 1.5^b^	66.0 ± 1.4^h^	9.7 ± 0.4^bc^	7.7 ± 0.4^f^	47.0 ± 0.9^cd^	39.5 ± 0.7^f^	6.4 ± 0.3^b^	3.6 ± 0.1^e^
**L9**	80.0 ± 1.5^d^	68.5 ± 1.0^g^	10.5 ± 0.5^a^	8.0 ± 0.3^e^	51.5 ± 1.0^a^	43.0 ± 0.7^e^	6.5 ± 0.2^b^	4.6 ± 0.1^d^
**Giza 138**	92.5 ± 3.0^a^	75.5 ± 1.8^e^	10.5 ± 0.6^a^	7.5 ± 0.3^f^	48.5 ± 0.7^c^	39.0 ± 0.8^f^	5.5 ± 0.2^c^	4.7 ± 0.1^d^

Mean values and standard deviation (± SD) in each column followed by a different lower-case letter (a, b, bc, c, cd, d, f, e, ef, f, fg, h) are significantly different according to Duncan's multiple range tests at *p* ≤ 0.05

The data in Table 5 shows that salinity stress significantly reduced plant height across all genotypes. The longest genotypes were Giza 138 and L6, with values of 92.5 and 91.0 cm under normal conditions and 75.5 and 73.0 cm, respectively, under saline conditions. In contrast, L1 shows the genotypes that are the shortest in both normal and salt conditions, with values of 74.0 cm and 65.0 cm, respectively.

The L4 and L6 genotypes exhibited the highest numbers of grain spike^−1^ under normal conditions (52.5 and 52.05, respectively) and salt-stressed conditions (40.0 and 40.0). Salt stress negatively influenced the number of grain spike^−1^. Under salt stress, L1 displayed the lowest number of grain spike^−1^ with average values of (46.0 and 33.0), respectively. Additionally, grain yield (g) in all barley genotypes was significantly impacted by salt stress. The results showed that under stressful conditions, L4, Giza 138, L6, and L9 had the highest grain yield values (4.9, 4.7, 4.7, and 4.6 g), while, L1 showed the lowest readings (2.5 g).

### Effect of salinity stress on tolerance indices

The ratio of yield under stress to yield under non-stress conditions is considered the stress susceptibility index (SSI). According to SSI index, genotypes that have an index smaller than one are considered to be stress tolerant. On the other hand, genotypes that have stress index greater than one are susceptible.

The results in Table 6 showed that the 10 barley genotypes under investigation varied in how they responded to salt stress. The genotypes L2, L5, and Giza 138 could be regarded as moderately tolerant genotypes because they have SSI values (1.05 and 110, respectively) that are lower than those of genotypes L4, L6, L9, and Giza 138 (0.44, 0.36, 0.77, and 0.46, respectively). In contrast, L1 has the highest SSI value (1.74), making it the most susceptible genotype.

**Table 6:** Salinity tolerance indices for barley genotypes based on grain yield per plant

Genotypes	SSI	STI	MP	TOL	YI	YSI
L1	1.74	0.055	4.1	3.1	0.062	0.45
L2	1.05	0.162	5.0	2.0	0.099	0.67
L3	1.28	0.187	5.3	2.7	0.096	0.59
L4	0.44	0.220	5.3	0.8	0.121	0.86
L5	1.10	0.125	4.7	2.0	0.091	0.65
L6	0.36	0.175	5.0	0.6	0.116	0.89
L7	1.29	0.153	5.0	2.6	0.091	0.59
L8	1.37	0.149	5.0	1.6	0.089	0.56
L9	0.77	0.286	5.7	2.8	0.121	0.75
Giza 138	0.46	0.188	5.1	0.8	0.116	0.85

*SSI* Stress susceptibility index, *STI* Stress tolerance index, *MP* Mean productivity, *TOL* Tolerance index, *YI* Yield index, *YSI* Yield stability index

Genotypes L4, L9, and Giza 138 have a higher stress tolerance index (STI) (0.220, 0.286, and 0.188 respectively) and mean productivity (MP) (5.3, 5.7, and 5.1 respectively), whereas genotype L1 has a lower STI (0.055) and MP (4.1). L1 had the highest tolerance index (TOL) value, indicating that these genotypes were more sensitive to salt and experienced a greater reduction in grain yield (GY) under salt stress conditions. In contrast, L4, L6, and L9, as well as Giza 138, had the lowest TOL values, indicating that these genotypes were tolerant and experienced a lower GY reduction under stress conditions. The Giza 138, L4, L6, and L9 genotypes also showed the greatest yield index (YI) and yield stability index (YSI) values, whereas L1 genotype was the most sensitive and had the least YI and YSI values.

### Biochemical components

#### Effect of salinity stress on osmolytes

The data in Table 7 shows that all barley genotypes that were subjected to salt stress had considerably higher levels of total soluble sugars and proline in their shoots than plants grown under normal conditions. L4, L6, L9, and Giza 138 which are considered tolerant genotypes, showed the highest amounts of osmolytes (soluble sugar and proline). On the other hand, L1, which is considered a sensitive genotype, had the lowest values of osmolytes.

**Table 7:** Effect of salt stress on biochemical components of barley genotypes during two successive seasons (2020–2021 and 2021–2022)

Genotypes	Total soluble sugars (mg/g)	Proline (µmol/g FW)	Phenol (µg gallic acid /g DW)	Flavonoids (µg querectin/g DW)	Ascorbic acid (µg/g FW)	MDA (nmol MDA g^−1^ FW)	H_2_O_2_ (µmol g^−1^ FW)	Na + uptake (mg/g DW)	K + uptake (mg/g DW)	K + /Na + ratio
	**Control**									
**L1**	13.21 ± 0.1^e^	2.25 ± 0.01^h^	30.25 ± 0.3^i^	45.99 ± 0.5^f^	5.10 ± 0.1^h^	24.33 ± 0.6^b^	5.10 ± 0.04^b^	3.13 ± 0.01^bc^	2.13 ± 0.03^h^	0.68
**L2**	19.11 ± 0.3^c^	3.80 ± 0.03^f^	50.33 ± 0.4^ g^	55.22 ± 0.6^e^	7.20 ± 0.4^f^	16.56 ± 0.4^gh^	2.55 ± 0.02^h^	1.93 ± 0.02^f^	3.40 ± 0.04^e^	1.76
**L3**	16.15 ± 0.3^de^	4.80 ± 0.02^d^	64.69 ± 0.5^e^	64.54 ± 0.6^d^	5.15 ± 0.1^ h^	18.20 ± 0.7^ fg^	4.05 ± 0.04^de^	2.63 ± 0.01^d^	3.13 ± 0.03^f^	1.19
**L4**	19.00 ± 0.4^c^	5.45 ± 0.03^c^	80.22 ± 0.6^c^	75.20 ± 0.4^c^	11.10 ± 0.2^c^	12.40 ± 0.2^i^	3.09 ± 0.03^g^	1.43 ± 0.02^ h^	4.03 ± 0.03^c^	2.82
**L5**	15.21 ± 0.2^e^	4.15 ± 0.02^ef^	42.35 ± 0.4^ h^	45.66 ± 0.6^f^	8.18 ± 0.3^e^	17.10 ± 0.6^ g^	4.10 ± 0.02^d^	2.13 ± 0.01^e^	3.50 ± 0.03^e^	1.64
**L6**	27.50 ± 0.4^b^	4.75 ± 0.04^d^	85.76 ± 0.5^c^	75.20 ± 1.4^c^	11.25 ± 0.2^c^	12.20 ± 0.2^i^	2.60 ± 0.03^h^	1.93 ± 0.01^f^	4.73 ± 0.03^ab^	2.45
**L7**	19.10 ± 0.3^c^	3.80 ± 0.02^f^	55.25 ± 0.4^ fg^	55.24 ± 0.6^e^	7.18 ± 0.4^f^	15.56 ± 0.4^ h^	3.00 ± 0.03^g^	2.13 ± 0.02^e^	4.03 ± 0.02^ cd^	1.89
**L8**	16.22 ± 0.1^de^	3.55 ± 0.01^f^	65.60 ± 0.4^e^	63.51 ± 0.7^d^	8.55 ± 0.3^e^	20.40 ± 0.3^d^	3.30 ± 0.03^f^	2.63 ± 0.01^d^	3.13 ± 0.04^ef^	1.19
**L9**	20.25 ± 0.4^c^	5.30 ± 0.05^c^	70.32 ± 0.5^de^	86.30 ± 0.4^b^	8.88 ± 0.3^e^	9.88 ± 0.2^j^	2.15 ± 0.02^i^	1.43 ± 0.02^ h^	3.70 ± 0.03^d^	2.59
**Giza 138**	27.50 ± 0.2^b^	6.20 ± 0.04^b^	85.55 ± 0.5^c^	87.30 ± 0.4^b^	12.10 ± 0.3^b^	9.90 ± 0.2^j^	2.20 ± 0.02^i^	1.80 ± 0.01^ fg^	4.90 ± 0.02^a^	2.72
**Salt stress**										
**L1**	14.52 ± 0.1^e^	3.15 ± 0.02^ g^	40.20 ± 0.3^ h^	53.45 ± 0.6^e^	6.20 ± 0. 2^ g^	28.20 ± 0.5^a^	5.70 ± 0.04^a^	3.73 ± 0.01^a^	1.87 ± 0.02^i^	0.50
**L2**	25.12 ± 0.5^b^	4.55 ± 0.03^d^	60.10 ± 0.4^ef^	63.25 ± 0.3^d^	8.40 ± 0.2^e^	19.33 ± 0.3^e^	3.30 ± 0.02^f^	2.13 ± 0.01^e^	3.00 ± 0.03^ fg^	1.41
**L3**	17.25 ± 0.4^d^	5.08 ± 0.02^ cd^	75.30 ± 0.4^d^	74.67 ± 0.6^c^	6.55 ± 0.2^ g^	20.13 ± 0.5^de^	4.60 ± 0.03^c^	2.93 ± 0.01^c^	2.80 ± 0.03^ g^	0.96
**L4**	25.10 ± 0.6^b^	6.23 ± 0.03^b^	90.44 ± 0.6^bc^	86.30 ± 0.6^b^	12.25 ± 0.2^b^	15.40 ± 0.4^h^	3.50 ± 0.02^f^	1.63 ± 0.01^g^	3.87 ± 0.04^d^	2.37
**L5**	18.15 ± 0.3^ cd^	5.45 ± 0.04^c^	53.37 ± 0.4^g^	52.33 ± 0.4^e^	9.42 ± 0.4^d^	21.17 ± 0.5^c^	4.60 ± 0.04^c^	2.43 ± 0.02^d^	3.10 ± 0.02^f^	1.28
**L6**	39.30 ± 0.4^a^	5.30 ± 0.05^c^	93.20 ± 0.5^b^	85.34 ± 0.5^b^	12.40 ± 0.4^b^	15.40 ± 0.4^ h^	3.22 ± 0.03^ fg^	2.13 ± 0.01^e^	4.40 ± 0.02^b^	2.07
**L7**	25.00 ± 0.4^b^	4.40 ± 0.02^de^	60.55 ± 0.5^e^	66.44 ± 0.3^d^	8.30 ± 0.3^e^	19.23 ± 0.3^ef^	3.32 ± 0.02^f^	2.53 ± 0.02^d^	3.83 ± 0.03^d^	1.51
**L8**	17.10 ± 0.1^d^	4.20 ± 0.03^e^	75.30 ± 0.4^d^	72.68 ± 0.5^c^	9.66 ± 0.4^d^	22.00 ± 0.5^c^	3.71 ± 0.03^e^	2.93 ± 0.01^c^	2.80 ± 0.03^ g^	0.96
**L9**	26.04 ± 0.7^b^	6.55 ± 0.04^b^	81.20 ± 0.5^c^	94.35 ± 0.5^a^	9.79 ± 0.4^d^	12.52 ± 0.4^i^	2.55 ± 0.01^ h^	1.73 ± 0.01^ g^	3.43 ± 0.03^e^	2.24
**Giza 138**	40.20 ± 0.5^a^	7.50 ± 0.05^a^	103.10 ± 0.7^a^	94.55 ± 0.5^a^	13.45 ± 0.4^a^	12.42 ± 0.3^i^	2.55 ± 0.01^ h^	2.00 ± 0.01^e^	4.62 ± 0.02^b^	2.31

Mean values and standard deviation (± SD) in each column followed by a different lower-case letter (a, b, bc, c, cd, d, de, f, e, ef, f, fg, h, i, j) are significantly different according to Duncan's multiple range tests at *p* ≤ 0.05

#### Effect of salinity stress on secondary metabolites and ascorbic acid

The data in Table 7 shows that all barley genotypes significantly increased their total phenol, total flavonoid, and ascorbic acid contents under salt stress in contrast to control plants. L4, L6, L9, and Giza 138 had the highest levels of total phenol, total flavonoid, and ascorbic acid content, while L1 had the lowest levels.

#### Effect of salinity stress on oxidative stress

Salinity stress caused the accumulation of MDA and H_2_O_2_ in all barley genotypes as compared to non-stressed plants. In addition, as indicated in Table 7, L1 accumulated higher amounts of MDA and H_2_O_2_ than the other genotypes, while L4, L6, L9, and Giza 138 accumulated lower amounts of the same substances.

#### Effect of salinity stress on mineral contents

The barley genotypes responded differentially to mineral concentrations when exposed to salt stress. Compared to unstressed plants, the concentration of Na^+^ increased significantly in all barley genotypes, while the amount of K^+^ decreased in all barley genotypes (Table 7). In all barley genotypes, the K^+^/Na^+^ ratio significantly decreased. Moreover, the tolerant genotypes (L4, L6, L9, and Giza 138) had the greatest K^+^/Na^+^ ratio and the lowest Na^+^ value, whereas L1 exhibited the opposite pattern.

#### SDS-PAGE of seed storage protein patterns

**Fig. 1:**
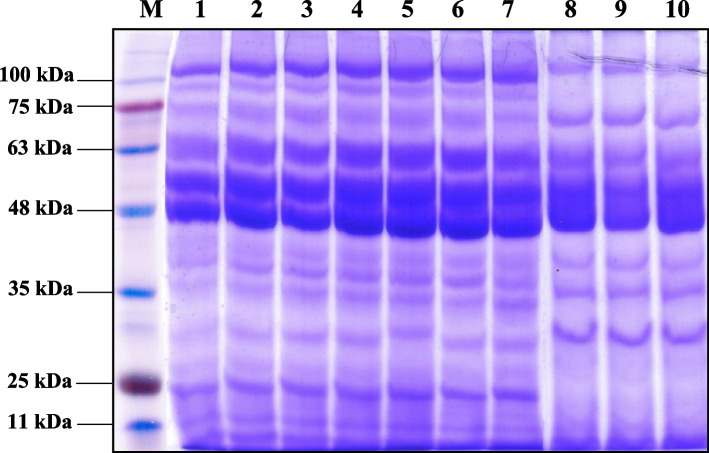
SDS banding pattern of seed protein for the ten barley genotypes tested under salt stress. M (protein marker), Lanes 1–10: L1, L2, L3, L4, L5, L6, L7, L8, L9 and Giza138 genotypes

Figure 1 shows the electrophoretic banding pattern of the harvested seeds of ten genotypes of barley. Table 8 provides information on SDS-PAGE scanning, scoring bands, their molecular masses in kDa, and whether bands are present (1) or absent (0). The 10 barley genotypes have 19 bands with a polymorphism rate of 42.11%. The polypeptides had a molecular weight that varied from 240.7 to 8.4 kDa. The L2 and L3 genotypes had the most bands, which totaled 17, while the L8 and L9 genotypes had the fewest, which totaled 11. Moreover, 11 monomorphic bands and 8 polymorphic bands make up the resulting profile. No distinct bands were identified. Only lines 6 and Giza 138 contained the molecular mass band (18.5 KDa). L6 and L7 also displayed a band of molecular mass (15.0 KDa).

**Table 8:** The molecular mass (Mr.) in kilo-Daltons (kDa) of the produced SDS-PAGE of seed protein bands and their presence (1) or absence (0), number and type of the bands as well as the percentage of the polymorphism detected in the ten barley genotypes under salt stress conditions

Band No	Rf	Mr (kDa.)	Genotypes										
			**1**	**2**	**3**	**4**	**5**	**6**		**7**	**8**	**9**	**10**
1	0.089	240.67	1	1	1	0	0	0		0	0	0	0
2	0.108	208.33	1	1	1	0	0	0		0	0	0	0
3	0.128	176.33	1	1	1	0	0	0		0	0	0	0
4	0.168	122.50	1	1	1	1	1	1		1	1	1	1
5	0.208	90.29	1	1	1	1	1	1		1	0	0	0
6	0.269	71.70	1	1	1	1	1	1		1	1	1	1
7	0.356	61.00	1	1	1	1	1	1		1	1	1	1
8	0.437	51.90	1	1	1	1	1	1		1	1	1	1
9	0.498	46.10	1	1	1	1	1	1		1	1	1	1
10	0.575	40.67	0	1	1	1	1	1		1	0	0	0
11	0.606	38.2	1	1	1	1	1	1		1	1	1	1
12	0.656	34.9	1	1	1	1	1	1		1	1	1	1
13	0.759	30.00	1	1	1	1	1	1		1	1	1	1
14	0.825	26.30	1	1	1	1	1	1		1	1	1	1
15	0.872	22.71	1	1	1	1	1	1		1	0	0	0
16	0.899	18.50	0	0	0	0	0	1		0	0	0	1
17	0.918	15.00	0	0	0	0	0	1		1	0	0	0
18	0.939	11.70	1	1	1	1	1	1		1	1	1	1
19	0.971	8.40	1	1	1	1	1	1		1	1	1	1
Total			16	17	17	14	14	16		15	11	11	12
Total number of bands		Monomorphic bands			Polymorphic bands						Polymorphism (%)		
					Shared Bands				Unique Bands				
19		11			8				0		42.11		

### Molecular marker

#### SCoT analysis

**Fig. 2:**
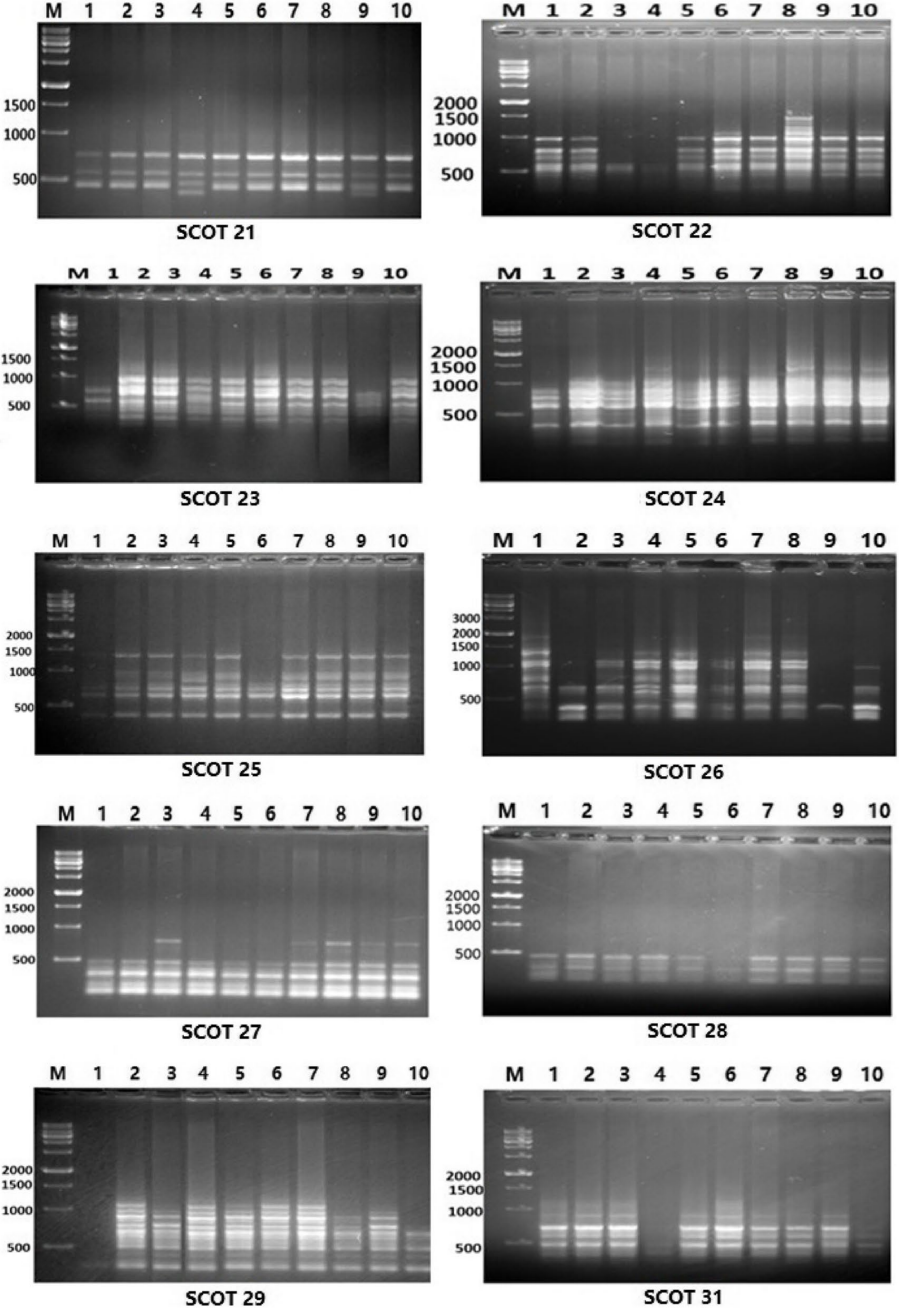
SCoT fingerprints of the ten barely genotypes tested using ten primers. Lanes 1–10: L1, L2, L3, L4, L5, L6, L7, L8, L9 and Giza138 genotypes

As shown in Fig. 2 and Table 9, ten SCoT primers produced a total of 94 fragments, 29 of which were monomorphic. The remaining 65 bands were polymorphic, with 62.18% (polymorphism) including 21 distinct bands (9 positive specific markers and 12 negative specific markers). The molecular size ranged from 200 to 1000 bp, and the number of fragments was between 4 and 14. In addition, SCoT-22 produced 14 bands, followed by SCoT-23, SCoT-26, and SCoT-29 which produced 12 bands, and SCoT-24 and SCoT-32 which produced 11 bands.

**Table 9:** Band variation and polymorphism percentage for the ten barley genotypes using SCoT and ISSR primers

Primer	No of bands	Polymorphic bands	Monomorphic bands	Polymorphism %	Unique bands	ve +	ve-	Genotype	Band size
SCOT primers									
SCOT-21	4	1	3	25.0	0	0	0	0	
SCOT- 22	14	12	2	85.7	4	4	0	8	1200, 1300, 1400, 1500
SCOT- 23	12	8	4	66.7	4	(770)1	(400, 850, 900) 3	1,4,9	400, 770, 850, 900
SCOT-24	11	7	4	63.6	3	(1500)1	(500, 1000)2	8,4,1	500, 1000, 1500
SCOT-25	7	4	3	57.1	1	0	1	6	1400
SCOT -26	12	11	1	91.7	3	0	3	9,8,9	300, 550, 600
SCOT-27	6	1	5	16.7	0	0	0	0	0
SCOT-28	5	3	2	60.0	1	0	1	6	500
SCOT-29	12	11	1	91.7	3	0	3	1	400, 500, 700
SCOT-31	11	7	4	63.6	3	3	0	6	950, 1000, 1100
Total	**94**	**65**	**29**	**62.18**	**22**	**9**	**13**		
ISSR primers									
ISSR-807	8	7	1	87.5	4	0	4	4	600, 700, 800, 1000
ISSR-810	9	1	8	11.1	0	0	0	0	0
ISSR-835	8	4	4	50.0	2	0	2	10	900, 1000
ISSR-841	10	3	7	30.0	0	0	0	0	0
UBC826	3	0	3	0.0	0	0	0	0	0
UBC827	7	6	1	85.7	3	(1000)1	(200, 300)2	10	200, 300, 1000
UBC835	9	2	7	22.2	0	0	0	0	0
Total	**54**	**23**	**31**	**40.9**	**9**	**1**	**8**		

Additionally, the primers SCoT-22 (12), SCoT-26, and SCoT-29, which produced 11 polymorphic bands, had the highest number of polymorphic bands. The SCoT-22 and SCoT-23 primers produced the greatest number of bands (four). Additionally, the SCoT-21 and SCoT-27 primers produced the fewest distinct bands (0) and the fewest polymorphism fragments (1). In addition, SCoT-26 and SCoT-29 primers showed the highest polymorphism rate (91.7%), followed by SCOT-22 (85.7%) and SCoT-23 (66.7%), while SCoT-27 primer showed the lowest polymorphism rate (16.7%).

The data in Table 9 was successful in revealing the molecular genetic differences among the 10 distinct barley genotypes, establishing specific markers for each genotype as a basis for classification. These bands can also be thought of as molecular genetic markers for salt stress tolerance in these 10 genotypes of barley.

The primer SCoT-22 exhibited four positive specific markers with molecular sizes of 1200, 1300, 1400, and 1500 bp for L8. By using the primer SCoT-23, four markers were produced, one positive and three negative. The positive marker had a molecular size of 770 bp and was found in L4, while the negative markers had sizes of 400, 800, and 900 bp and were found in L9. In this regard, three distinct markers were produced for the primer SCoT-24, one positive marker for L8 with a molecular size of 1500 bp and two negative markers for L4 with a molecular size of 500 bp and L1 with a molecular size of 1000 bp. Concerning primer SCsoT-25, one negative marker was observed by this primer with a molecular size of 1400 bp for L6. The primer SCoT-26 exhibited three negative specific markers with molecular sizes of 300 and 600 bp in L9, and 550 bp in L8. The primer SCoT-28 exhibited one negative specific marker for the L6 with a molecular size of 500 bp. The primer SCoT-29 L1 produced three negative specific markers with molecular sizes of 400, 500, and 700 bp. In addition, the SCoT-31 primer produced three positive particular markers for L6 with molecular sizes of 900, 1000, and 1100 bp.

### ISSR analysis

**Fig. 3:**
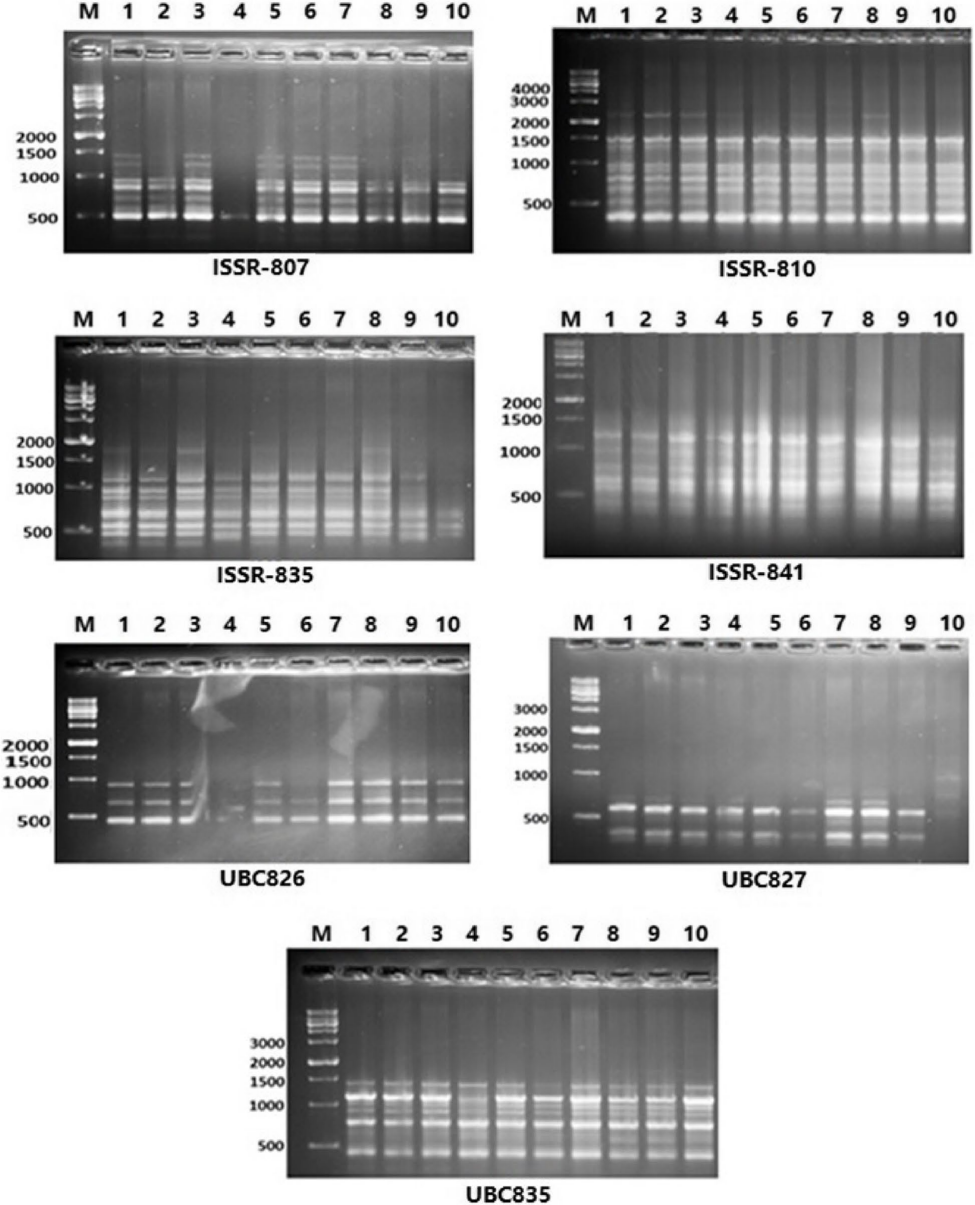
ISSR fingerprints of the ten barely genotypes tested using seven primers. Lanes 1–10: L1, L2, L3, L4, L5, L6, L7, L8, L9 and Giza138 genotypes

Table 9 and Fig. 3 provide an overview of the degree of polymorphism and contrast the ability of ISSR markers to discriminate between different genotypes. Seven ISSR primers were used, and a total of 54 amplified bands (31 monomorphic and 23 polymorphic) were found. Their molecular sizes ranged from 300 to 2200 bp.

The UBC827 and ISSR-807 primers had the highest polymorphism values (85.7% and 0%, respectively), whereas ISSR-826 had the lowest polymorphism values. The ISSR-807 and UBC827 primers produced the most polymorphic bands about 7 and 6 bands, respectively. From ISSR primers, nine amplified bands (one positive and eight negative) were produced (Table 9). Using the ISSR-807 primer, the highest number of unique bands—four negative markers—were detected in L4. These bands had molecular weights of 600, 700, 800, and 1000 bp. ISSR-835 produced two negative unique markers with molecular sizes of 900 and 1000 bp in L10. The primer UBC827 produced three markers: one positive marker with a molecular size of 1000 bp was found in L10, and two negative markers with molecular sizes of 200 and 300 bp were also present in L10.

### Cluster analysis

**Fig. 4:**
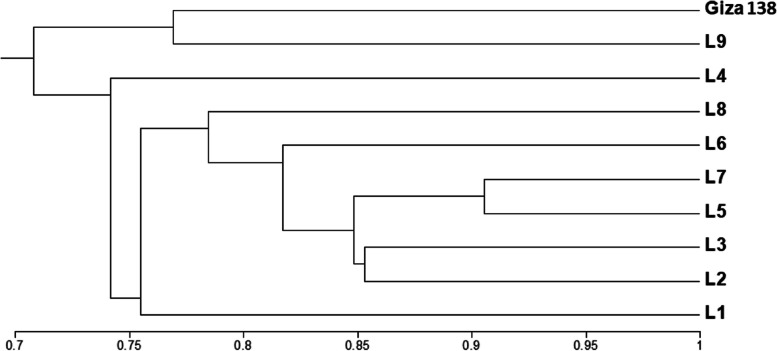
UPGMA cluster analysis based on Jaccard similarity coefficient, showing the genetic relationships among the ten barely genotypes tested, obtained from SCoT, ISSR and SDS-PAGE seed protein markers

Based on the SCOT, ISSR markers, and SDS-PAGE seed protein studies, Fig. 4 shows the dendrogram of the ten genotypes that were investigated. UPGMA cluster analysis revealed that the genotypes were classified into two main clusters. The first main cluster included Giza138 and L9 genotypes. The second main cluster included eight genotypes represented by a separate phenetic line that comprises the L4 genotype, and a sub-cluster comprises the other seven genotypes. Hence, this confirmed the high similarity between them. The levels of similarities among the ten genotypes tested based on the SCoT, ISSR, and SDS-PAGE seed protein profiles are illustrated in Table 10. According to the degree of similarity between the genotypes under study, the largest similarity between the L5 and L7 genotypes is 90.5%, while the lowest similarity between the L4 and Giza138 genotypes is 63.6%.

**Table 10:** Similarity matrix values generated using NTSYS software for the data produced from SCoT, ISSR and SDS-PAGE seed protein markers for the ten barely genotypes

	**L1**	**L2**	**L3**	**L4**	**L5**	**L6**	**L7**	**L8**	**L9**	**Giza138**
L1	1.000									
L2	0.793	1.000								
L3	0.776	0.853	1.000							
L4	0.678	0.755	0.776	1.000						
L5	0.788	0.876	0.871	0.788	1.000					
L6	0.704	0.800	0.784	0.682	0.854	1.000				
L7	0.764	0.813	0.833	0.788	0.905	0.830	1.000			
L8	0.704	0.753	0.761	0.727	0.830	0.738	0.842	1.000		
L9	0.674	0.764	0.701	0.686	0.748	0.711	0.760	0.759	1.000	
Giza138	0.648	0.690	0.664	0.636	0.711	0.698	0.723	0.757	0.769	1.000

### Principal component analysis (PCA)

**Fig. 5:**
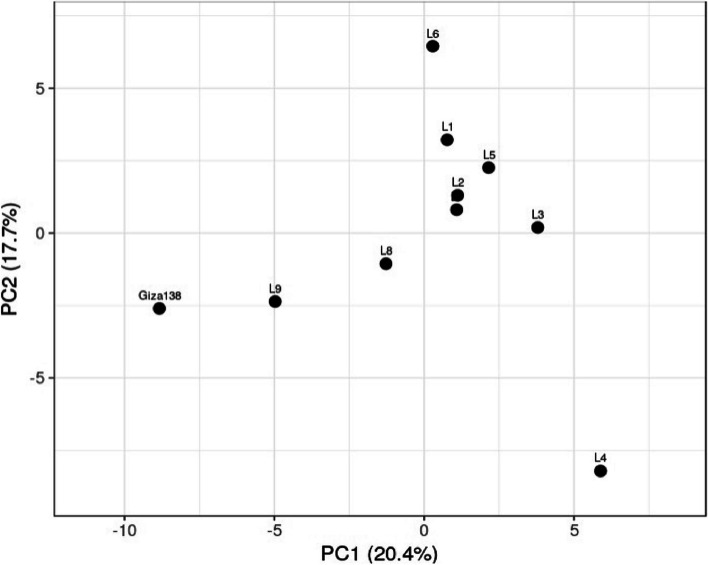
An illustration of the genetic diversity expressed in 10 Egyptian barley genotypes, according to a principal component analysis (PCA) based on polymorphism of SCoT and ISSR markers, using PAST software

The genetic diversity of the studied genotypes was assessed using the genetic diversity parameter data from SCoT and ISSR markers, as well as multivariate clustering, PCA, and Heatmap analysis. In a PCA scatter plot, the SCoT and ISSR markers demonstrate the markers' reliability in identifying the tested genotypes. The barley genotypes L1, L2, L3, L5, L6, and L7 were shown to be unique from the other genotypes using PCA analysis (Fig. 5). Additionally, there was evidence of neighboring affinity between the genotypes L8, L9, and Giza 138. On the other hand, L4 was dispersed far from one another.

### Multivariate heatmap

**Fig. 6:**
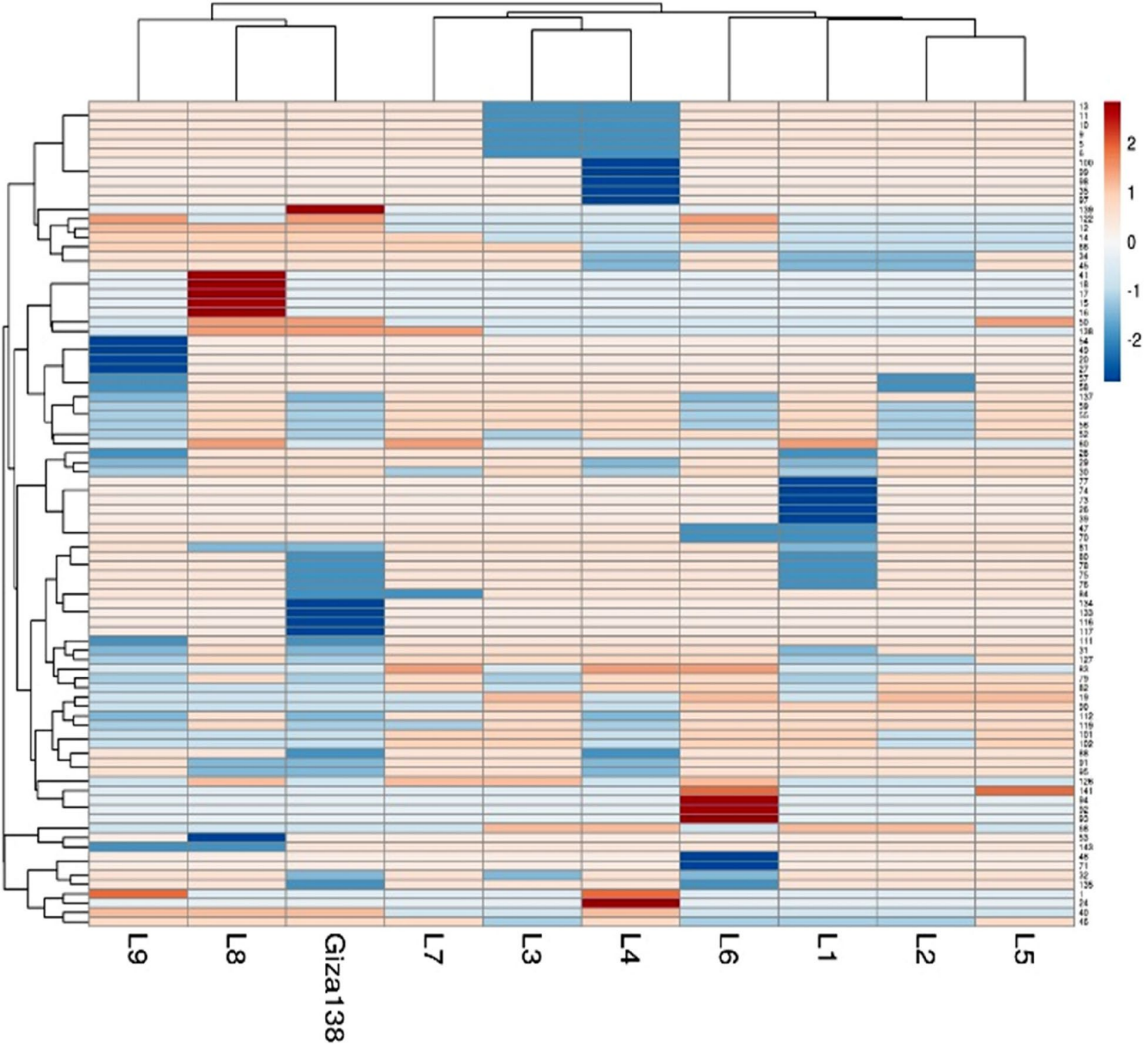
Multivariate heatmap illustrating the genetic diversity of 10 Egyptian barley genotypes, based on 10 SCoT primers and 7 ISSR primers for using the module of a heatmap of ClustVis—an online tool for clustering and visualizing of multivariate data

A Heatmap provides detailed information about the genetic variation of plant breeds, and multivariate compound similarity analysis is typically used to reveal further information about this genetic variance. A Heatmap created by ClustVis—an online tool for clustering and visualizing of multivariate data compound similarities. Ten Egyptian barley genotypes were grouped into four clusters, as shown by the columns, with at least two genotypes in each cluster (Fig. 6). The genotypes L9, L8, and Giza 138 were all part of the first cluster. The genotypes L7, L3, and L4 were distinguished as two adjacent genotype pairs. L6 and L1 made up the third cluster, and L2 and L5 were two neighboring clusters that made up the fourth cluster (Fig. 6).

## Discussion

By 2050, the population will have increased to around ten billion, which will result in an increase in agricultural demand of at least 50% given that agriculture now uses 69% of the freshwater withdrawn globally. The amount of water resources may not be enough to meet human demand in the ensuing decades [55]. Farmers are thus obliged to use sewage water or semi-saline subsurface water [56]. Plants are harmed by salinization in several ways; including water stress, nutritional problems, and ion toxicity that affects photosynthesis. By lowering the soil's water potential and causing water deficits, salinity reduces plants' capacity to absorb water. All plant growth stages are disrupted due to the salt-specific effects of increased sodium (Na^+^) and chloride (Cl^−^) concentrations in tissues caused by ion toxicity. Ion-specific damage was primarily caused by Na^+^, which has a major impact on both protein synthesis and enzyme activation. Plant physiological systems are impacted by salinity stress, which also changes plant development, mineral distribution, membrane instability, and photosynthetic production [57].

The best indicator for assessing the effects of different abiotic stresses on plants is plant growth. Salinity inhibited plant height, spike length, number of grains per spike, and grain production per plant (g) in the current study when compared to control plants in all barley genotypes. In contrast to salt-tolerant genotypes, the decline was more pronounced in salt-sensitive genotypes. In comparison to unstressed plants, the L1 barley genotype showed the most dramatic reductions in plant height (12.2%), spike length (30.9%), number of grains per spike (28.3%), and grain yield perplant (55.4%). In comparison to other genotypes, this genotype is thought to be sensitive to salinity stress. Under salinity stress, the other genotypes were deemed tolerable and moderately tolerant. Mariey et al. [58] who confirmed that the decrease in grain yield was caused by a decrease in yield components, reported similar results. According to Mariey et al. [58], grain yield is the result of the action and interaction of numerous environmental, agronomical, and physiological variables. High NaCl content lowers plant water potential, which lowers water flow into fruit and slows the rate of fruit expansion, which could account for the decreased yield [59]. Additionally, these decreases in growth under salinity stress may have been brought on by the osmotic effect of the saline solutions, which disrupted cell elongation, cell division, and enlargement, DNA replication in interphase, water balance, and nutritional imbalance, ultimately reducing photosynthesis and slowing growth rates [1].

Since the genotypes L4, L6, L9, and Giza 138 had the lowest SSI and TOL values, we can infer that they are the most salt-tolerant genotypes. In contrast, L1 was thought to be the sensitive genotype because it exhibited the highest SSI and TOL values. The genotypes L4, L6, L9, and Giza 138 had the highest values for STI, MP, YI, and YSI, while L1 had the lowest values for these traits. According to similar research by Hamam and Negim [60], SSI was frequently utilized by scientists to categorize sensitive and resistant genotypes. The low TOL genotypes, on the other hand, were thought to be most tolerant genotypes. Additionally, MP was employed by Ghonaim et al. [11] as a resistance standard for wheat genotypes exposed to low levels of stress. Additionally, the higher STI readings of up to 1 demonstrate a high level of stress tolerance. The genotype's increased salt tolerance and increased yield are reflected in the genotype's raised STI rate, as reported by Ghonaim et al. [11].

The total soluble sugar, proline, total phenols, total flavonoids, and ascorbic acid were all significantly higher under salinity stress, according to analysis of the analyzed biochemical features. Our findings showed that under conditions of salt toxicity, salt-tolerant barley genotypes accumulated significantly more total soluble sugars, proline, total phenols, total flavonoids, and ascorbic acid than salt-sensitive genotypes. Under adverse conditions, the soluble sugar is essential for maintaining membrane integrity and adjusting osmotic pressure. Under salt stress, rice's total soluble sugar considerably increased and was closely correlated with cultivars' capacity for osmotic adjustment [61]. Proline accumulation is one of the most important physiological indicators of salt tolerance [62]. In order for plants to modify their osmotic potential, the buildup of organic molecules under salt stress is crucial. One class of hydrophilic macromolecules is proline. The function of proline in osmotic control is controversial. According to the theory of Ashraf et al. [61], the buildup of proline was crucial for wheat cultivars to respond to osmotic pressure and have improved salt tolerance. According to a different theory, proline served as a ROS scavenger and stabilizer of membrane structures rather than directly reducing osmotic stress [61]. Because they have phenolic hydroxyl groups that are active, phenolic components have considerable antioxidant ability. Reactive oxygen species are created in cells and tissues when plants are subjected to salinity stress, therefore the plants produce phenolics and flavonoids to combat salt stress [63]. Through direct action or enzyme catalysis, ascorbic acid is essential for quenching intermediate or excited reactive forms of oxygen. In addition, Sen et al. [64], found that ascorbic acid directly scavenges ROS and uses an ascorbate peroxidase process to convert H_2_O_2_ into water. Sarker et al. [65] found that the antioxidant ascorbate plays a crucial role in controlling the homeostasis of ROS in plants and reducing the effects of oxidative stress.

According to Ashraf et al. [61], salt toxicity causes an excessive amount of ROS like hydrogen peroxide and MDA to be produced, which damages vital cellular parts like nucleic acids, proteins, and membranes. Malondialdehyde (MDA), a by-product of membrane lipid peroxidation, is used to quantify ROS-induced damage to membranes [11]. In contrast to salt-tolerant genotypes, MDA and H_2_O_2_ levels substantially rose under salt toxicity in salt-sensitive genotypes in our study. It appears that salt tolerance is associated with less active lipid peroxidation since the sensitive genotypes of barley (L9) contain higher levels of MDA than the tolerant genotypes.. These findings are in line with those of Ashraf et al. [61], who discovered that salt toxicity caused salt-sensitive wheat cultivars to substantially raise their MDA and H_2_O_2_ levels. The salt-tolerant cultivars, on the other hand, displayed a non-significant rise in MDA levels during salt stress, indicating only minor oxidative damage to cellular membranes and other components.

The increased Na^+^ accumulation in plant cells results in ion toxicity. Under salt stress, K^+^ is crucial for maintaining ion homeostasis and controlling plant growth and development [66]. In the current investigation, salt stress was observed to considerably increase Na^+^ buildup while decreasing K^+^ and K^+^/Na^+^. The K^+^/Na^+^ ratio is higher in the tolerant genotypes. Our results are consistent with those of Ghonaim et al. [11], who suggested that wheat genotypes with a higher K^+^/Na^+^ proportion could be viewed as salt-tolerant genotypes grown in saline conditions. The other genotypes could be considered sensitive genotypes since they contain more Na^+^ and have a lower K^+^/Na^+^ ratio.

The ten barley genotypes under study have a seed protein pattern with 19 bands and a polymorphism percentage of 42.11%. The number of bands on the more tolerable genotypes ranged from 14 to 17. The research of Ghonaim et al. [11], who found that the protein contents of bread wheat varied depending on genotypes and environmental factors, can be used to support these inconsistent protein profiles. Regarding this, an increase in the synthesis of particular protein sets (new bands) acting as molecular chaperones may be linked to protein accumulation at low molecular weights under saline stress. A variety of cellular processes, including protein synthesis and degradation, macromolecular assembly and disassembly, maintaining proteins in their native state and preventing their aggregation under stress, and controlling their cellular compartments, are carried out by the diverse group of proteins known as molecular chaperones [67]. The entire suppression of the protein synthesis genes caused by stress may account for the absence of some polypeptides. As a result, the created tissues had lost their capacity to produce these proteins while under stress. Additionally, it's likely that the stress-related impairment of the genes and incomplete recovery of the inhibition mean that they are not entirely silent [11]. This increases the ability of biochemical genetic markers to identify genotypes with greater environmental stress tolerance. It can be inferred that the acquired results may be beneficial for interspecific hybridization, breeding programs, and taxonomic definition of diverse accessions.

Understanding how species and genotypes adapt to abiotic environments, which result in changes in the genetic makeup of these plants, requires knowledge of genetic variability. Several studies have employed a variety of molecular markers to evaluate the genetic diversity of barley [67]. A useful genetic technique for analysing the links between barley genotypes is ISSR markers [67]. In this study, 10 SCoT primers in particular showed a total of 94 fragments, 29 of which were monomorphic, and 65 bands, or 62.18% (polymorphism), of which 21 were unique bands (9 positive specific markers and 12 negative specific markers). For examining the genetic divergence between barley types, prior investigations used SCoT markers, which showed between 66.67 and 100% polymorphism [68, 69].

Understanding population diversity is necessary for properly utilizing the genetic variety that is available to breeders [70, 71]. Therefore, the fundamental benefit of genotype differentiation at the molecular level is to give future breeders molecular insights while also decreasing selection costs for breeding programmers by logically describing the linkages between genotypes. Seven ISSR primers produced a total of 54 amplified bands, of which 31 were monomorphic and 23 had 40.9% polymorphism. In many other plants, ISSR-PCR DNA analysis has been utilized to successfully categorize genetic relationships and provide appropriate markers for molecular data to examine genetic diversity. The findings also point to a significant potential for ISSR to discover DNA-based variation among genotypes of the same species. As well as serving as molecular fingerprints for the tested genotypes, these identified polymorphic bands can be thought of as possible markers to identify salinity-tolerant genotypes or cultivars for marker assisted selection (MAS) in barley water-deficient resistance breeding programs.

UPGMA cluster analysis based on the SDS-PAGE seed protein profile and molecular markers pool data divided the ten genotypes into two main clusters at 0.71 genetic similarity coefficient level. The first main cluster included Giza138 and L9 genotypes. The second main cluster included eight genotypes represented by a separate phenetic line that comprises L4 genotype and a sub-cluster comprises the other seven genotypes. According to the degree of similarity between the genotypes under study, the largest similarity between the L5 and L7 genotypes is 90.5%, while the lowest similarity between the L4 and Giza138 genotypes is 63.6%. Many authors used the combination of different biochemical and molecular marker systems to evaluate the genetic similarity among individuals or populations [22, 72, 73].

The findings from SCoT and ISSR markers evaluated in this work may have been influenced by factors such as genotype production, the instability of TNB insertion events, and behavior under environmental conditions, and this is in agreement with Guasmi et al. [74]. There may be a link between genotype variety and the high level of polymorphism shown in ISSR markers [22]. The genotypes were divided into four groups that were more closely related to their usage in Egypt, despite variances in the PCA results based on molecular data. Heatmaps provide a wealth of information about the genetic diversity of plant breeds [14].

## Conclusion

The study suggests that four barley genotypes (L4, L6, L9, and Giza 138) adapted to salt stress conditions. On the other hand, L1 genotype was the most susceptible one. Stress susceptibility index (SSI) can be utilized as a useful selection criterion for determining genotype salt tolerance for grain yield. High levels of genetic variation among barley genotypes were shown by the SDS-PAGE of seed proteins. Additionally, SCoT and ISSR successfully identified molecular genetic markers between different genotypes and labeled each genotype with distinct bands. In addition, L4, L6, L9, and Giza 138 have the ability to grow in soil that contains salinity water or is irrigated with saline water. We recommended using L4, L6, L9, and Giza 138 as potential varieties and employing these lines as parents in breeding programes due to their high productivity under salt stress according to salinity tolerance indices illustrated in Table 6.

### Supplementary Information

**Additional file 1.**

## Data Availability

The datasets during and/or analysed during the current study available from the corresponding author on reasonable request.
