# Supplementary figures and images for: Agro-morphological, biochemical, and molecular markers of barley genotypes grown under salinity stress conditions
Source: BMC Plant Biol. 2023 Oct 30;23:526. doi: 10.1186/s12870-023-04550-y (PMC10614329; doi:10.1186/s12870-023-04550-y)

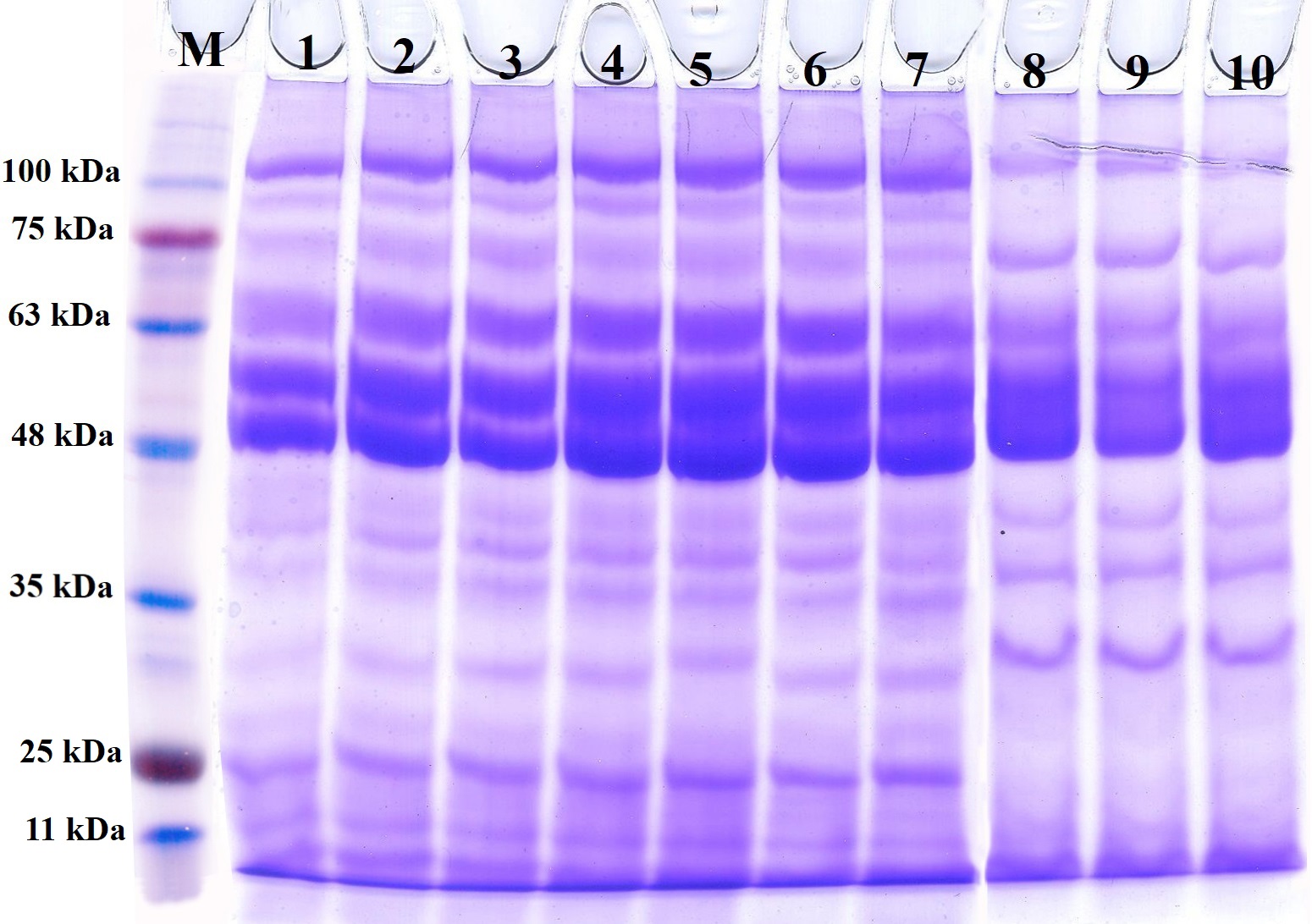

Supplement: Supplementary file 1 — Additional file 1. [file 12870_2023_4550_MOESM1_ESM.jpg]
